# Adrenomedullin Improves Hypertension and Vascular Remodeling partly through the Receptor-Mediated AMPK Pathway in Rats with Obesity-Related Hypertension

**DOI:** 10.3390/ijms24043943

**Published:** 2023-02-15

**Authors:** Hong-Yu Wang, Fang-Zheng Wang, Rui Chang, Qian Wang, Si-Yu Liu, Ze-Xiong Cheng, Qing Gao, Hong Zhou, Ye-Bo Zhou

**Affiliations:** Department of Physiology, Nanjing Medical University, Nanjing 211166, China

**Keywords:** adrenomedullin, obesity-related hypertension, vascular smooth muscle cells, inflammation, oxidative stress, calcification

## Abstract

Adrenomedullin (ADM) is a novel cardiovascular peptide with anti-inflammatory and antioxidant properties. Chronic inflammation, oxidative stress and calcification play pivotal roles in the pathogenesis of vascular dysfunction in obesity-related hypertension (OH). Our study aimed to explore the effects of ADM on the vascular inflammation, oxidative stress and calcification in rats with OH. Eight-week-old Sprague Dawley male rats were fed with either a Control diet or a high fat diet (HFD) for 28 weeks. Next, the OH rats were randomly subdivided into two groups as follows: (1) HFD control group, and (2) HFD with ADM. A 4-week treatment with ADM (7.2 μg/kg/day, ip) not only improved hypertension and vascular remodeling, but also inhibited vascular inflammation, oxidative stress and calcification in aorta of rats with OH. In vitro experiments, ADM (10 nM) in A7r5 cells (rat thoracic aorta smooth muscle cells) attenuated palmitic acid (PA, 200 μM) or angiotensin II (Ang II, 10 nM) alone or their combination treatment-induced inflammation, oxidative stress and calcification, which were effectively inhibited by the ADM receptor antagonist ADM22-52 and AMP-activated protein kinase (AMPK) inhibitor Compound C, respectively. Moreover, ADM treatment significantly inhibited Ang II type 1 receptor (AT1R) protein expression in aorta of rats with OH or in PA-treated A7r5 cells. ADM improved hypertension, vascular remodeling and arterial stiffness, and attenuated inflammation, oxidative stress and calcification in OH state partially via receptor-mediated AMPK pathway. The results also raise the possibility that ADM will be considered for improving hypertension and vascular damage in patients with OH.

## 1. Introduction

Obesity is a major risk factor for cardiovascular diseases (CVD), which increased morbidity and mortality in the world [1]. Obesity-related hypertension (OH) is closely associated with chronic heart failure (CHF) and stroke partly due to vascular abnormalities such as vascular remodeling and the increased arterial stiffening [2]. Inflammation, oxidative stress and calcification have emerged as critical mediators of OH-associated vascular damage [2]. The atherogenic dyslipidemia, proinflammatory state and the overactivation of renin-angiotensin-aldosterone system (RAAS) have been linked to OH [3]. Therefore, the improvement of vascular abnormalities caused by OH may prevent the development of CVD associated with obesity.

Normal vascular smooth muscle cells (VSMCs) are highly differentiated cells within the medial layer of blood vessels. They perform a critical role in regulating blood pressure and vascular function, but their dysfunction contributes to vascular pathologies in OH [4]. The migration, proliferation and phenotype transition (from a contractile phenotype to a synthetic phenotype) of VSMCs are the core processes in vascular remodeling, which can be influenced by inflammation and oxidative stress. If this phenotypic switching is excessive, it will promote the increases of extracellular matrix synthesis and calcification of VSMCs that further participate in vascular remodeling and the increase in stiffness and reduce the elasticity of artery wall leading to the development of occlusive vascular diseases, such as postangioplasty restenosis, atherosclerosis, hypertension and aneurysm formation [4,5]. 

The cardiovascular risk factors RAAS and free fatty acids (FFAs), such as the main saturated fatty acid palmitic acid (PA), increase the incidence of vascular abnormalities in subjects with OH [4,6,7]. High levels of circulating angiotensin II (Ang II) and PA are known to be important triggers for vascular damage during the development of OH [4,5]. They can also promote oxidative stress, inflammation and calcification in VSMCs [8,9,10,11,12].

Adrenomedullin (ADM) is an endogenous active peptide with many functions, including tissue repair, anti-inflammatory and antioxidant effects, and organ protection [13,14,15]. It was expected to be a candidate therapeutic agent for CVD, including CHF [15]. ADM and ADM receptors are highly expressed in vascular tissues, and plasma ADM level is markedly increased in people with obesity [16,17,18] indicating that it may be critically involved in the pathogenesis of OH. 

At present, the potential effects and mechanisms of ADM to treat OH, especially in blood vessels, have not been thoroughly explored. Our recent work has found that ADM could improve cardiac remodeling and function in rats with OH [19]. Therefore, it is better to explore the effects and molecular mechanisms linking ADM to vascular pathophysiology in OH, and to assess the therapeutic targets that may be of specific benefit in combating vascular diseases in OH. 

## 2. Materials and Methods 

### 2.1. Animals

Eight-week-old male Sprague Dawley rats (300~350 g) were randomly assigned to Control group (*n* = 8), fed a Control diet (fat provided 12% kilocalories, TROPHIC Animal Feed High-tech Co Ltd., Nantong, China) and high fat diet (HFD, fat provided 45% kilocalories, TROPHIC Animal Feed High-tech Co Ltd, Nantong, China) group (*n* = 37). Rats were housed and permitted access to tap water and diet (Control diet or HFD) ad libitum in a room with a 12 h light/dark cycle and controllable temperature and humidity for 28 weeks. The experiments were approved by the animal research ethics committee of Nanjing Medical University (1911016, 14 September 2020) and conformed to the Guidelines for the Care and Use of Laboratory Animals (NIH publication, eighth edition, 2011). The criterion for the OH rats was that the body weight (BW) was 120% higher than that of the mean weight of control rats, and the systolic blood pressure (SBP) was more than or equal to 140 mmHg after 28-week HFD feeding. OH rats (*n* = 17) were further randomized into two groups and continued to receive the HFD for 4 weeks. The intraperitoneal injection of ADM (7.2 μg/kg/day) was applied to the OH rats (*n* = 9), and the remaining rats (*n* = 8) received an equal volume of saline. At the end of 32 weeks, the BW and SBP were measured in conscious state. The heart and visceral white adipose tissue (WAT) were removed under anesthesia by using sodium pentobarbital and then weighted. 

### 2.2. Measurement of SBP and Heart Rate 

The SBP of tail artery in waking state were measured by a computerized tail-cuff system (NIBP, ADInstruments, Sydney, NSW, Australia). The rat was trained for SBP detection to obtain a steady pulsation of tail artery before the formal experiment began as previously described [19]. In a rat, the obtained values were analyzed by averaging 10 measurements.

### 2.3. Aortic Samples Preparation

Under anesthesia, the whole aortic tissue of rat was removed quickly. One part of the aortic tissue was kept at −80 °C for further analysis, and the other was incubated in formalin for 8 h. The sections of aortic tissue slices (6~8 μm) were used for hematoxylin-eosin (H&E), dihydroethidium (DHE) and alizarin red staining, respectively. 

### 2.4. Alizarin Red Staining

For evaluating calcification, aortic slides were dehydrated and rinsed in distilled water, and the cultured A7r5 cells (rat thoracic aorta smooth muscle cells) grown in 6-well plates were fixed in 4% paraformaldehyde for 15 min after washing with PBS three times. The alizarin red solution (pH 4.2, 1%) was used to incubate the aortic slides or A7r5 cells for 3–5 min at room temperature and it was washed with distilled water. Finally, the staining was observed by microscopy. 

### 2.5. Measurement of Plasma Insulin and Triglyceride 

The tail vein blood was collected for the measurement of fasting insulin and triglyceride levels. Plasma was obtained by centrifugation of blood samples at 900× *g* for 10 min at 4 °C with a centrifuge (MicroCL 17R, Thermo Fisher Scientific Inc., Waltham, MA, USA). The plasma level of insulin was determined by Enzyme-linked immunosorbent assay (Elisa) by using a kit (USCN Business Co., Ltd., Wuhan, China) referring to the manufacturer’s instructions. The triglyceride level was detected by colorimetric assay with a kit (Jiancheng Bioengineering, Nanjing, China) according to the manufacturer’s descriptions. The optical density at a certain wavelength was measured by a microplate reader (ELX800, BioTek, Winooski, VT, USA).

### 2.6. Cell Culture and Treatment

A7r5 cells were cultured in Dulbecco’s modified Eagle’s medium (DMEM) containing 10% fetal bovine serum (FBS), 100 U/mL penicillin and 100 μg/mL streptomycin in a 37 °C incubator with 5% CO_2_. For ADM treatment, the cells were treated with 10 nM ADM for 30 min, then treated with PA or Ang II alone or their combination for 24 h (for detection of inflammation and oxidative stress-related markers) or 7 days (for detection of calcification-related markers). For ADM receptors antagonizing or the inhibition of signaling pathway, 1 μM ADM22-52 or 10 μM AMPK inhibitor Compound C was added into the medium for 30 min before the ADM pretreatment, respectively. 

### 2.7. Cell Viability Assay

The A7r5 cells suspension (100 μL) was added into a 96-well plate and cultured in a cell incubator at 37 °C for 24 h. The effect of PA (200 μM), Ang II (10 nM) or ADM (10 nM) alone on cell viability was detected by Cell Counting Kit-8 (CCK8) cell cytotoxicity test referring to the manufacturer’s instructions as previously described [19]. 

### 2.8. DHE Fluorescence Staining for ROS Level Assay

Reactive oxygen species (ROS) production in aortic tissue or A7r5 cells was detected with DHE staining as previously described [19]. Briefly, the tissue sections (7 μm) or the A7r5 cells (3 × 10^5^ cells/mL) in six-well plates were incubated with 10 μM DHE in PBS for 30 min in a dark and humidified container at 37 °C. Then, they were rinsed with cold PBS for three times and were observed by a fluorescence microscopy (DP70, Olympus Optical, Tokyo, Japan). 

### 2.9. Determination of ROS Level and NADPH Oxidase Activity

The enhanced lucigenin-derived chemiluminescence method was adopted to measure the nicotinamide adenine dinucleotide phosphate (NADPH) oxidase activity and ROS level in aortic tissues or A7r5 cells. Both dark-adapted 100 μM NADPH and 5 μM lucigenin were used to trigger the photon emission and the background chemiluminescence was detected by a luminometer (Turner, CA, USA). The data were presented as the mean of light unit (MLU)/min/mg protein. 

### 2.10. Measurement of Calcium Content 

Calcium content of the aortic tissues or A7r5 cells was determined by O-cresolphthalein colorimetric (OCPC) method by using a kit (Nanjing Jiancheng Bioengineering, Nanjing, China). The aortic tissues or A7r5 cells were decalcified with HCl at 37 °C for 24 h. The mixed working reagent solution containing OCPC, 8-hydroxyquinoline and ethanolamine buffer were added into the supernatant fluid. The solutions were incubated at 30 °C for 5 min. The absorbance of the compound solution was determined at 600 nm wavelength.

### 2.11. Measurement of Alkaline Phosphatase (ALP) Activity

ALP activity was measured by using an ALP assay kit (Nanjing Jiancheng Bioengineering, Nanjing, China). The proteins were extracted from the aortic tissues or A7r5 cells in 0.05% Triton X-100 in PBS and quantified using a bicinchoninic acid (BCA, ThermoFisher, Waltham, MA, USA) protein assay. The 5 μL supernatant of samples was mixed with reaction mixture including alkaline buffer solution 50 μL mainly containing disodium phenyl phosphate and substrate solution 50 μL mainly containing 4-aminoantipyrine and potassium cyanide. They were incubated at 37 °C for 15 min, then developer 150 μL was added into each well. The absorbance was detected at 520 nm wavelength and the results were normalized to the level of total protein. 

### 2.12. Western Blot Analysis

Aortic tissues or A7r5 cells were homogenized in RIPA lysis buffer. After protein quantification, equal quantities of tissues or cells protein lysates were separated by polyacrylamide gel electrophoresis (PAGE) (Bio-Rad), then were electrotransferred to a nitrocellulose membrane as previously described [19]. The membrane was incubated with the primary antibody against α-actin (1:3000), Runt-related transcription factor 2 (Runx2, 1:1000), tumor necrosis factor-α (TNF-α, 1:1000), interleukin-1β (IL-1β, 1:1000), IL6 (1:1000), NOX2 (1:1000), NOX4 (1:1000), ADM (1:1000), calcitonin receptor-like receptor (CRLR, 1:1000), receptor activity modifying protein 2 (RAMP2, 1:1000), RAMP3 (1:1000) or GAPDH (1:3000), respectively, overnight at 4 °C, then secondary antibody (horseradish peroxidase-conjugated anti-rabbit or anti-mouse) for 1 h. The polyclonal antibodies against α-actin, Runx2, ADM, CRLR, RAMP2, RAMP3, IL-1β, IL6, NOX2 and NOX4 were generated by immunizing rabbits and GAPDH monoclonal antibody was generated by immunizing mouse. Band densities were captured by using Odyssey Imaging System (LI-COR Biosciences, Lincoln, Nebraska). Finally, the protein level was evaluated by Image J software and normalized to GAPDH protein expression level.

### 2.13. Reagents and Antibodies 

Rat ADM (molecular formula: C_242_H_381_N_77_O_75_S_5_) was obtained from Bachem (Bubendorff, Switzerland). PA and Compound C were purchased from Sigma Aldrich (St. Louis, MO, USA). ADM22-52 was purchased from Anaspec (Fremont, CA, USA). DMEM, 0.25% trypsin-EDTA, fetal bovine serum, trypsin and streptomycin/penicillin were from Thermo Fisher Scientific (Waltham, MA, USA). The primary antibodies against α-actin and Runx2 were purchased from Abcam (Burlingame, CA, USA). ADM, CRLR, RAMP2, RAMP3, TNF-α and IL6 were obtained from Affinity Biosciences (Pottstown, PA, USA). The antibodies of IL-1β, NOX2, NOX4 and GAPDH were from Proteintech (SANYING, Wuhan, China). 

### 2.14. Statistics

The GraphPad Prism version 8.0.2 (San Diego, CA, USA) software was used in this study to analyze all data. Shapiro–Wilk test were used to evaluate normality of the data. Homogeneity of variances was assessed by the *F* test or Brown–Forsythe test. All data showed normal distribution and passed equal variance testing. Statistical analyses between two groups with normal distribution were performed using a two-tailed unpaired *t*-test as indicated in figure legends. One-way or two-way ANOVA followed by Bonferroni post hoc analysis was employed in the analyses of more than two groups with normal distribution as indicated in figure legends. The data were shown as mean ± SEM. Statistical significance was accepted at *p* < 0.05.

## 3. Results

### 3.1. The Effects of ADM on Body Weight, Metabolic Parameters, Blood Pressure and Left Ventricular Mass in HFD-Induced OH Rats

**Figure 1:**
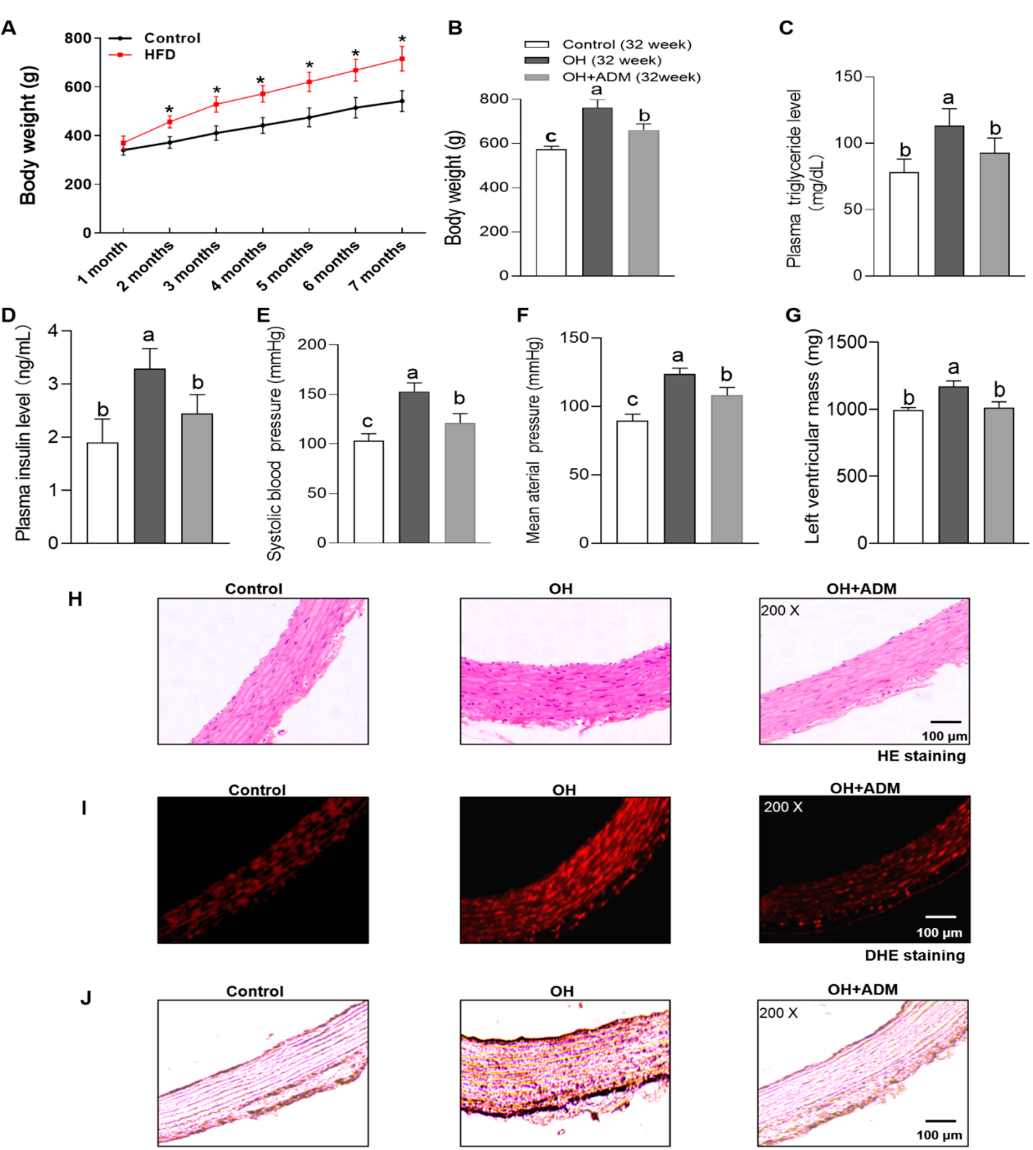
Body weight at the end of each month in rats fed with control diet or high fat diet (HFD) for 7 months (**A**) and the effects of Adrenomedullin (ADM, 7.2 μg/kg/day, ip) application for 4 weeks on body weight (**B**), plasma triglyceride and insulin levels (**C**,**D**), systolic blood pressure (**E**), mean arterial pressure (**F**), left ventricular mass (**G**), vascular remodeling ((**H**), HE staining), reactive oxygen species (ROS) level ((**I**), DHE fluorescence staining) and calcification ((**J**), alizarin red staining) in the tunica media of aorta after 32 weeks of HFD feeding. OH: obesity-related hypertension. The Control and OH rats were treated with saline (vehicle). *n* = 5–8 rats for each group. Values are presented as mean ± SEM. * *p* < 0.05 vs. Control (A, two-way ANOVA followed by Bonferroni post hoc analysis). Values with the same superscript letter are not significantly different and the different letters indicate significant differences between the groups (*p* < 0.05, one-way ANOVA followed by Bonferroni post hoc analysis, (**B**–**G**)).

In this study, we found that HFD feeding for 32 weeks significantly led to obesity, hypertension (*p* < 0.0001, Figure 1A,B,E,F), myocardial hypertrophy (*p* < 0.0001, Figure 1G) and the increases of plasma triglyceride and insulin levels (*p* < 0.0001, Figure 1C,D), which were effectively inhibited by exogenous ADM application for 4 weeks (*p* < 0.0001 for Figure 1A,B,E–G, *p* = 0.0065 for Figure 1D, *p* = 0.0201 for Figure 1D). HFD feeding also significantly increased vascular remodeling, ROS level and calcification as demonstrated by H&E staining (Figure 1H), DHE staining (Figure 1I) and alizarin red staining (Figure 1J), respectively, which were markedly alleviated by ADM application in HFD-fed rats. Taken together, these results indicate that ADM can improve HFD-induced hypertension, vascular remodeling and the increase in vascular stiffness in obese state.

### 3.2. The Endogenous Expression of ADM and Its Receptor System and The Effects of ADM on Aortic Inflammation, Oxidative Stress and Calcification in HFD-Induced OH Rats

**Figure 2:**
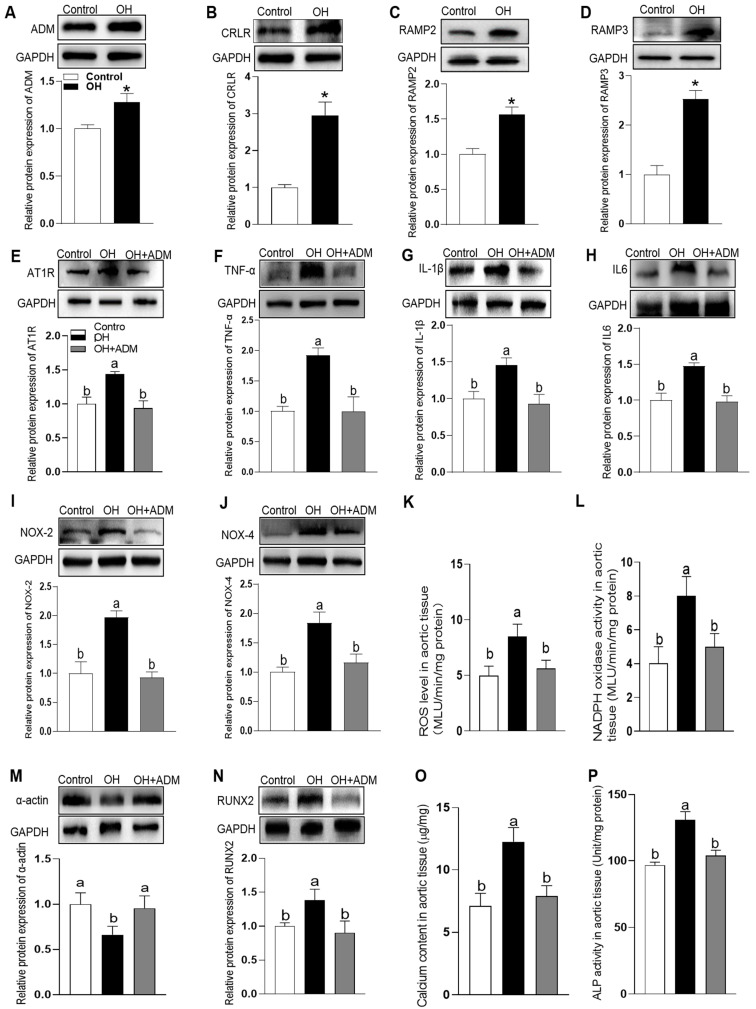
The endogenous protein expression levels of ADM, calcitonin receptor-like receptor (CRLR), receptor activity-modifying protein 2 (RAMP2) and RAMP3 (**A**–**D**) and the effects of ADM on AT1R expression (**E**), inflammation (**F**–**H**), oxidative stress (**I**–**L**) and calcification (**M**–**P**) in the tunica media of aorta after 32 weeks of high-fat diet (HFD) feeding. The protein expression levels of proinflammatory cytokines TNF-α (**F**), IL-1β (G) and IL6 (**H**) were measured to reflect inflammation; the two important catalytic subunits of NADPH NOX2 and NOX4 protein expression levels (**I**,**J**), ROS level (**K**) and NADPH oxidase activity (**L**) were detected to reflect oxidative stress; and the smooth-muscle lineage marker α-actin and runt-related transcription factor 2 (RUNX2, a DNA-binding transcription factor that regulates target genes in bone formation) protein expression levels (**M**,**N**), calcium content (**O**) and ALP activity (**P**) were determined to reflect calcification. NADPH, nicotinamide adenine dinucleotide phosphate. GAPDH was used as an internal control for Western blotting analysis. *n* = 4–8 rats. The values are presented as the mean ± SEM. * *p* < 0.05 vs. control group (two-tailed unpaired *t*-test, (**A**–**D**)). Values with the same superscript letter are not significantly different and the different letters indicate significant differences between the groups (*p* < 0.05, one-way ANOVA followed by Bonferroni post hoc analysis, (**E**–**P**)).

Western blot results showed that the endogenous protein expressions of ADM and its receptor system including CRLR, RAMP2 and RAMP3 were significantly increased in the tunica media of aorta in OH rats when compared to the control rats (*p* = 0.0014, *p* < 0.0001, *p* = 0.0002, *p* < 0.0001, respectively. Figure 2A–D). The results suggest that ADM and its receptor system may play important roles in regulating the function of VSMCs under OH state. In the vasculature, OH is associated with substantial inflammatory and oxidative responses, which mediate the pathogenesis of vascular injury and remodeling [20]. We examined the protein expressions of pro-inflammatory cytokines in tunica media of aorta. The results in OH rats showed that TNF-α, IL-1β and IL6 protein levels were significantly up-regulated when compared to the control rats (*p* < 0.0001, *p* = 0.0065, *p* = 0.0011, respectively. Figure 2F–H), which were effectively reduced by ADM application when compared to OH rats (*p* < 0.0001, *p* = 0.0031, *p* = 0.0009, respectively. Figure 2F–H). NADPH oxidase is a dominant enzyme to catalyze the formation of ROS, which plays a role in the development of CVD [21]. In this study, we examined the ROS level (Figure 2K), NADPH oxidase activity (Figure 2L) and its two important catalytic subunits NOX2 and NOX4 protein expressions (Figure 2I,J) to reflect the oxidative stress state. They all had a significant increase in aorta of OH rats compared to the control rats (all *p* < 0.0001), and ADM administration significantly decreased their levels compared to OH rats (*p* < 0.0001, *p* = 0.0003, *p* = 0.0002, and *p* = 0.0003, respectively. Figure 2I–L). These results suggest that ADM is involved in the improvement of OH-induced vascular inflammation and oxidative stress. Both inflammation and oxidative stress can induce the osteogenic transition of VSMCs which promotes the occurrence of vascular calcification resulting in the increased vascular stiffness [22]. In this study, we also examined the calcification-related indicators, namely calcium content, ALP activity, protein expressions of Runx2, a transcription factor essential for bone formation and α-actin, a phenotypic marker of smooth muscle cells, in aorta of HFD-induced OH rats. Compared to the control rats, the results showed that there were marked increases in the calcium content, ALP activity and the protein expression of Runx2 (*p* < 0.0001, *p* < 0.0001, and *p* = 0.0483, respectively. Figure 2N–P), but the α-actin protein expression was significantly reduced (*p* = 0.0007. Figure 2M). These changes were remarkably reversed by ADM administration compared to OH rats, (*p* < 0.0001, *p* < 0.0001, and *p* = 0.0175, respectively. Figure 2N–P; and *p* = 0.0023. Figure 2M) suggesting that ADM can effectively improve vascular stiffness involving the inhibition of arterial medial calcification and osteoblastic phenotypic transition of VSMCs in OH state.

### 3.3. The Effects of Exogenous ADM Pretreatment on PA-Induced Cell Viability, Inflammation and Oxidative Stress in A7r5 Cells and Its Possible Mechanisms

**Figure 3:**
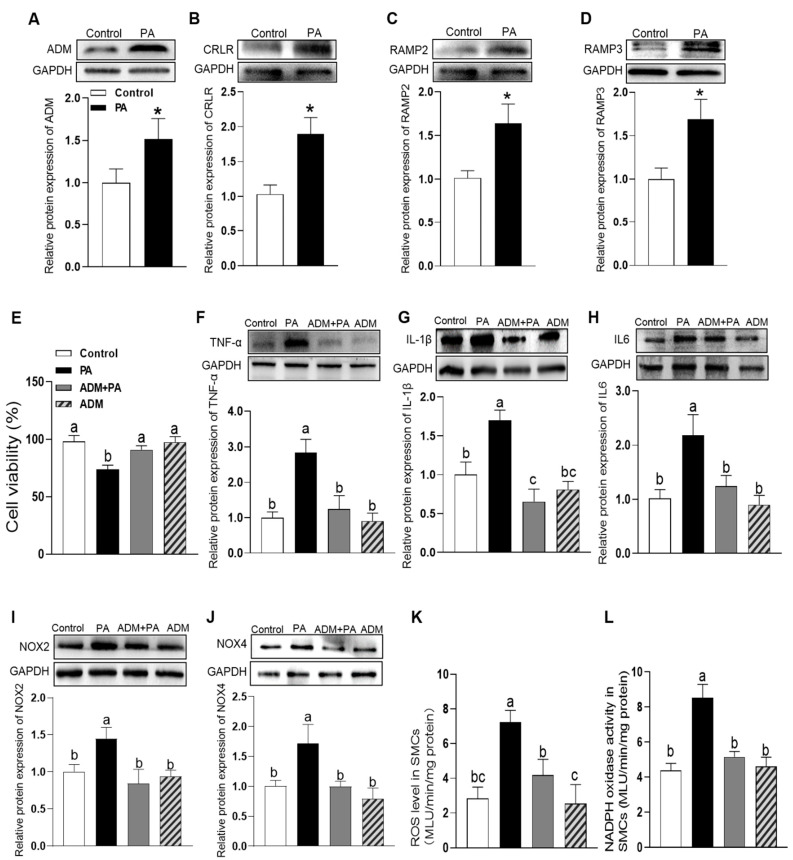
The endogenous protein expression levels of ADM, CRLR, RAMP2 and RAMP3 in A7r5 cells (**A**–**D**) exposed to 200 μM PA for 24 h. The effects of ADM (10 nM) on cell viability (**E**), inflammation (**F**–**H**) and oxidative stress (**I**–**L**) stimulated by PA. The protein expression levels of proinflammatory cytokines TNF-α (**F**), IL-1β (**G**) and IL6 (**H**) were measured to reflect inflammation, and the NOX2 and NOX4 protein expression levels (**I**,**J**), ROS level (**K**) and NADPH activity (**L**) were detected to reflect oxidative stress. The A7r5 cells were pretreated with 10 nM ADM for 30 min subsequently treated by PA for 24 h. *n* = 3–6. Each value indicates mean ± SEM. * *p* < 0.05 vs. control group (two-tailed unpaired *t*-test, (**A**–**D**)). Values with the same superscript letter are not significantly different, and the different letters indicate significant differences between the groups (*p* < 0.05, one-way ANOVA followed by Bonferroni post hoc analysis, (**E**–**L**)).

**Figure 4:**
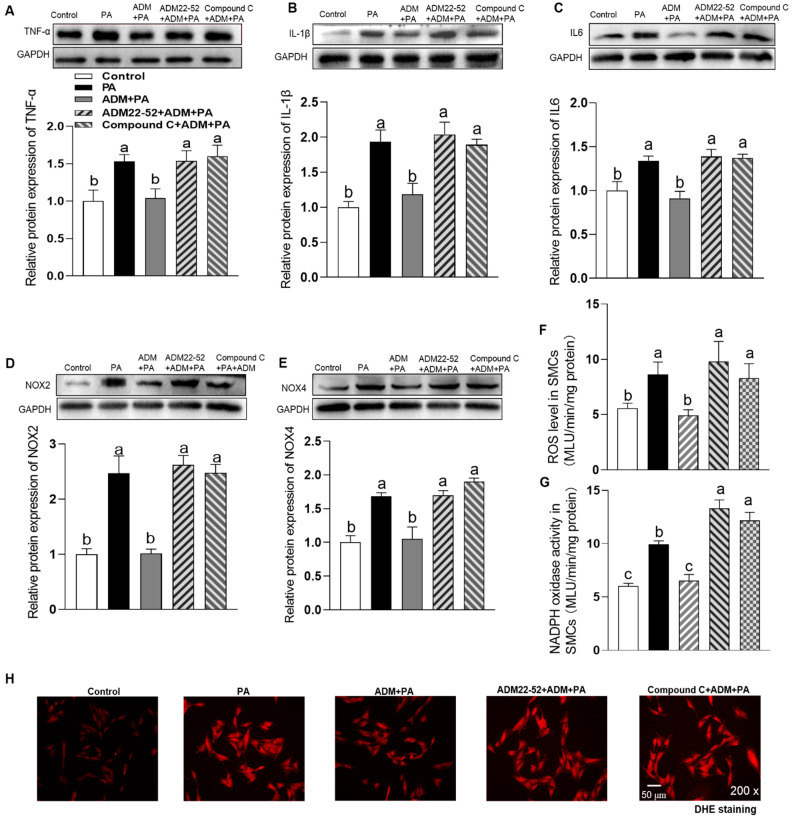
The effects of ADM receptor antagonist ADM22-52 (1 μM) or AMPK activation inhibitor compound C (10 μM) on ADM’s response to inflammation and oxidative stress induced by PA (200 μM) in A7r5 cells. The protein expression levels of proinflammatory cytokines TNF-α (**A**), IL-1β (**B**) and IL6 (**C**) were measured to reflect inflammation state and the NOX2 and NOX4 protein expression levels (**D**,**E**), ROS level (**F**), NADPH oxidase activity (**G**) and DHE fluorescence staining (intracellular ROS evaluation, **H**) were detected to reflect oxidative stress state. ADM (10 nM) was administrated in A7r5 cells for 30 min, then cells were exposed to PA for further 24 h. ADM22-52 or compound C was added 30 min before ADM application, respectively. Each value indicates mean ± SEM. *n* = 3 to 6. Values with the same superscript letter are not significantly different and the different letters indicate significant differences between the groups (*p* < 0.05, one-way ANOVA followed by Bonferroni post hoc analysis, (**A**–**G**)).

Elevated FFAs levels are a common feature of obesity and perform an etiological role in the pathogenesis of some diseases. In particular, the saturated fatty acid PA, which makes up 30–40% of high plasma FFAs concentration, is a major contributor to vascular diseases. However, the roles and mechanisms of ADM in PA-induced damage in vascular smooth muscles cells (VSMCs) are not entirely clear. Therefore, PA was used in this study to mimic the high-saturated fatty acids environment of obesity. In A7r5 cells, the Western blotting results showed that PA (200 μM) treatment for 24 h induced the higher protein expressions of ADM, CRLR, RAMP2 and RAMP3 than those in the control group (*p* = 0.0119, *p* = 0.0054, *p* < 0.0001, and *p* = 0.0100, respectively. Figure 3A–D). CCK8 results showed that ADM alone did not produce significant changes in cell viability compared to the control group (*p* > 0.9999, Figure 3E), but PA alone significantly decreased it (*p* < 0.0001, Figure 3E) when compared to the control group. More importantly, ADM pretreatment obviously improved the cell viability in A7r5 cells (*p* < 0.0001, Figure 3E). We also observed that ADM in A7r5 cells significantly reduced the increased protein levels of PA-induced proinflammatory cytokines TNF-α, IL-1β and IL6 (all *p* < 0.0001, Figure 3F–H) and oxidative stress-related indicators, namely ROS level, NADPH activity, and NOX2 and NOX4 protein expressions (*p* < 0.0001, *p* = 0.0116, *p* = 0.0001, and *p* < 0.0001, respectively. Figure 3I–L) compared to the PA group. In order to explore the possible mechanisms of ADM’s action, we applied the ADM receptors antagonist ADM22-52 and AMP-activated protein kinase (AMPK) inhibitor Compound C in PA-treated cells, respectively. The results showed ADM22-52 or Compound C pretreatment could effectively attenuate the inhibitory roles of ADM in inflammation and oxidative stress caused by PA in A7r5 cells (Figure 4A–H. (ADM22-52: *p* = 0095, *p* < 0.0001, *p* = 0.0001, *p* < 0.0001, *p* = 0.0001, *p* < 0.0001, *p* < 0.0001, respectively. Figure 4A–G); (Compound C: *p* = 0040, *p* < 0.0001, *p* = 0002, *p* < 0.0001, *p* < 0.0001, *p* = 0004, and *p* < 0.0001, respectively. Figure 4A–G)) suggesting that ADM exerts anti-inflammatory and antioxidant effects in VSMCs under metabolic stress partly via receptor-mediated AMPK pathway.

### 3.4. The Effects of Exogenous ADM Pretreatment on PA Plus Ang II-Induced Inflammation and Oxidative Stress in A7r5 Cells and Its Possible Mechanisms

**Figure 5:**
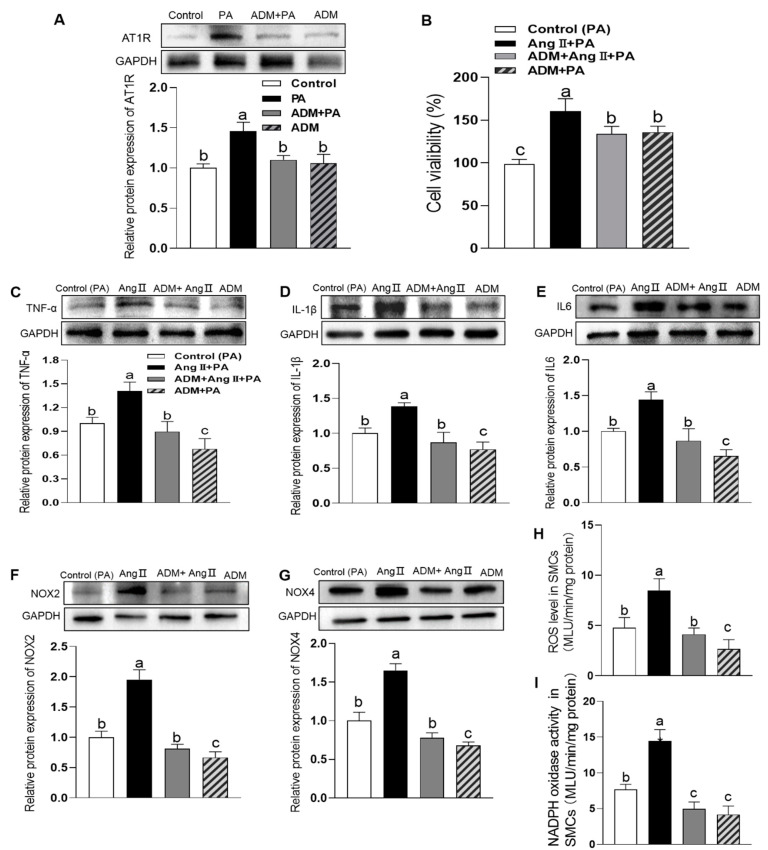
The effects of ADM (10 nM) on AT1R expression in A7r5 cells-treated by PA (200 μM, (**B**)), and on the combination of PA plus Ang II-stimulated cell viability (**A**), inflammation (**C**–**E**) and oxidative stress (**F**–**I**) in A7r5 cells. The protein expression levels of proinflammatory cytokines TNF-α (**C**), IL-1β (**D**) and IL6 (**E**) were measured to reflect inflammation state and the NOX2 and NOX4 protein expression levels (**F**,**G**), ROS level (**H**) and NADPH oxidase activity (I) were detected to reflect oxidative stress state. ADM was administrated in A7r5 cells for 30 min, then cells were exposed to PA plus Ang II (10 nM) for further 24 h. Each value indicates mean ± SEM. *n* = 3 to 6. Values with the same superscript letter are not significantly different and the different letters indicate significant differences between the groups (*p* < 0.05, one-way ANOVA followed by Bonferroni post hoc analysis).

**Figure 6:**
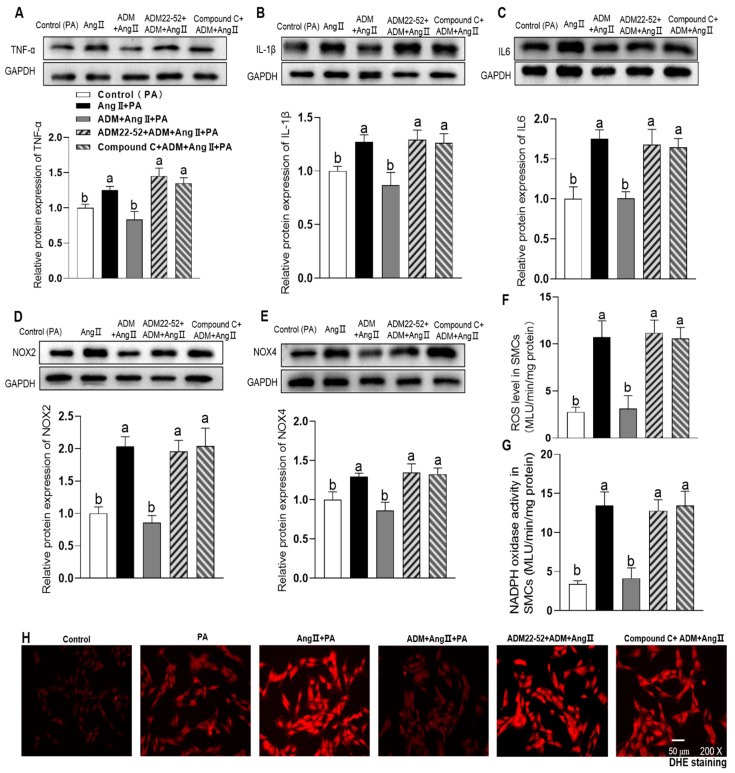
The roles of 1 μM ADM22-52 or 10 μM compound C in ADM’s response to the combination of PA plus Ang II-induced protein expressions of pro-inflammatory cytokines, including TNFα (**A**), IL-1β (**B**), IL-6 (**C**) and oxidative stress, including the protein expression of NOX2 (**D**) and NOX4 (**E**), ROS level (**F**), NADPH oxidase activity (**G**) and intracellular ROS evaluation by DHE fluorescence staining (**H**) in A7r5 cells. ADM (10 nM) was administrated in A7r5 cells for 30 min, then cells were exposed to PA plus Ang II (10 nM) for further 24 h. ADM22-52 or compound C was added 30 min before ADM application. Each value indicates mean ± SEM. *n* = 3 to 6. Values with the same superscript letter are not significantly different and the different letters indicate significant differences between the groups (*p* < 0.05, one-way ANOVA followed by Bonferroni post hoc analysis, (**A**–**G**)).

**Figure 7:**
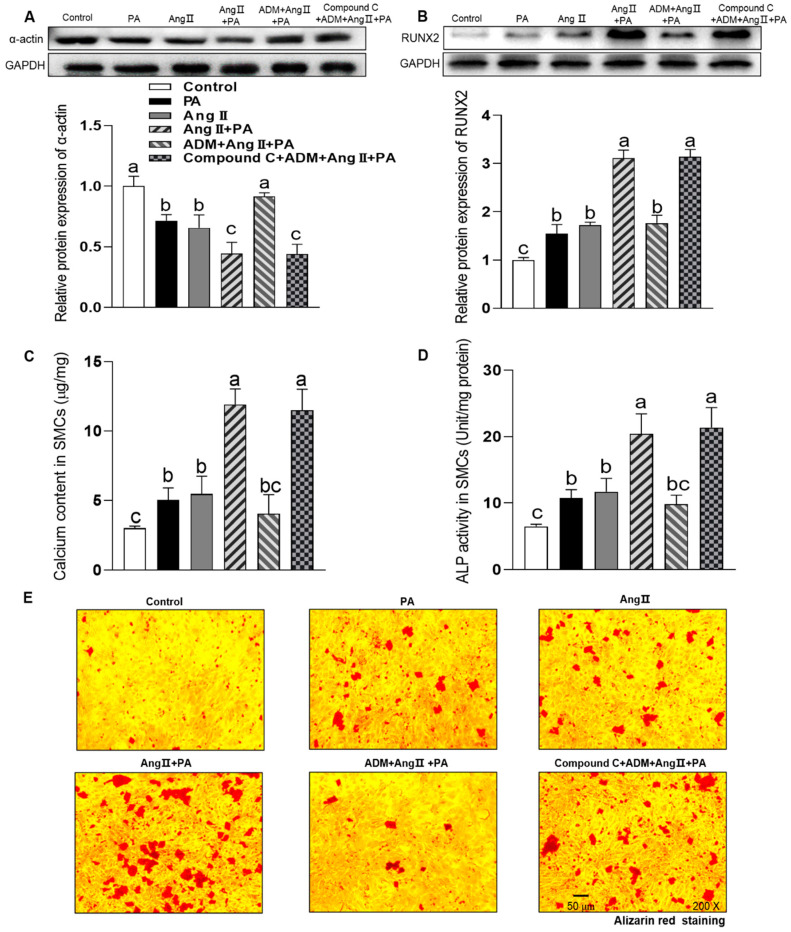
The effects of ADM on calcification in A7r5 cells treated by PA, Ang II or PA plus Ang II. AMPK activation inhibitor compound C (10 μM) was used to explored the related mechanisms of ADM’s action. The α-actin and RUNX2 protein expression levels (**A**,**B**), calcium content (**C**), ALP activity (**D**) and alizarin red staining (**E**, evaluating calcium deposition) were determined to reflect calcification. ADM (10 nM) was administrated in A7r5 cells for 30 min, then cells were exposed to PA (200 μM), Ang II (10 nM) or PA plus Ang II for another 7 days. Compound C was added 30 min before ADM application. Each value indicates mean ± SEM. N = 3 to 6. Values with the same superscript letter are not significantly different, and the different letters indicate significant differences between the groups (*p* < 0.05, one-way ANOVA followed by Bonferroni post hoc analysis, (**A**–**D**)).

Among the potential underlying pathophysiologic mechanisms of OH, overactivation of the RAAS also performs an important role [23]. Increased Ang II level is a major causative factor for obesity-related diseases, such as hypertension, myocardial hypertrophy, chronic kidney disease and so on [23]. Ang II contributes to inflammation, oxidative stress and calcification in the VSMCs [8,9,10]. In this study, we also explored the effects of ADM on the combination of PA plus Ang II-induced inflammation, oxidative stress and calcification. In aorta of OH rats, we found the marked increase in Ang II type 1 receptor (AT1R) protein expression (*p* = 0.0027, Figure 2E) which in A7r5 cells was also increased in culture with PA for 24 h (*p* = 0.0011, Figure 5A) compared to the control group. The results indicate that metabolic stress, namely elevated plasma levels of saturated FFAs, promotes the AT1R expression to enhance the pathogenic effects of Ang II on vascular damage. ADM administration notably recovered the cell viability abnormity caused by PA or PA plus Ang II (10 nM) in A7r5 cells (*p* < 0.0001, and *p* = 0.0008, respectively. Figure 5B). Ang II increased cell activity that may be related to the pro-proliferative effect. As we expected, PA plus Ang II induced more significant inflammation, namely the increased protein expression of proinflammatory cytokines TNF-α, IL-1β and IL-6 (all *p* < 0.0001, Figure 5C–E), and oxidative stress, namely increased ROS level, NADPH activity and protein expression of NOX2 and NOX4 (all *p* < 0.0001, Figure 5F–I) compared to the control group. These above changes were effectively reversed by ADM application (all *p* < 0.0001). Moreover, ADM receptors antagonist ADM22-52 (1 μM) and AMPK inhibitor Compound C (10 μM) pretreatment could also significantly inhibit the protective effects of ADM on PA plus Ang II-induced inflammation (*p* = 0.0011 (IL6), Figure 6C); (all *p* < 0.0001, Figure 6A,B,D–G), and oxidative stress Figure 6D–H; (all *p* < 0.0001, Figure 6D–G). Furthermore, ADM administration also effectively inhibited PA plus Ang II induced-calcification, and which were significantly reversed by ADM22-52 or Compound C. (Figure 7A–E; all *p* <0.0001, Figure 7A–D). These resuts suggests that receptor-mediated AMPK pathway may involve ADM’s anti-inflammatory, antioxidant and anti-calcification effects in OH state. 

## 4. Discussion

In this study, the primary novel findings were that the exogenous ADM application contributed to the improvement of vascular remodeling and stiffness, and lowered blood pressure. ADM alleviated inflammation, oxidative stress and calcification not only in the tunica media of aorta in rats with OH, but also in A7r5 cells-treated by PA. More importantly, we found ADM significantly attenuated the synergistic effects of PA and Ang II-induced inflammation, oxidative stress and calcification, which were partially associated with the receptor-mediated AMPK pathway.

ADM can participate in the regulation of many physiological functions and pathophysiological process of some diseases, such as vasodilation, angiogenesis, organ protection and tissue repair [24,25,26,27,28,29]. In our previous studies, ADM improved cardiac remodeling and function in rats with OH [19]. These findings suggest that ADM may have protective roles in vascular damage in OH state. Indeed, our results confirmed our speculation that exogenous ADM administration improved vascular remodeling and stiffness and decreased blood pressure in rats with OH. It also effectively reduced the protein expressions of proinflammatory factors and the related indicators of oxidative stress and calcification. Therefore, it is speculated that ADM improves vascular remodeling and stiffness partly through its anti-inflammatory, antioxidant and anti-calcific effects in OH-induced vascular injury. 

Metabolic stress in obesity state, especially excessive and saturated FFAs, such as PA, and the overactivity of the RAAS, specifically the vasoactive mediator Ang II, appear to be of particular importance in the genesis of vascular damage in OH [6,7,8,9,10,11,12]. In vitro, PA plus Ang II treatment induced more significant inflammation, oxidative stress and calcification. More importantly, ADM application could notably attenuate the synergistic pathogenic effects that suggests ADM may be a more effective peptide for the treatment of vascular diseases in people with OH.

Cardiovascular tissues have highest density of ADM receptors and binding sites [24,25]. In this study, we found that there were higher expression of ADM and its receptor system in aorta of OH rats and their expressions were also upregulated by PA in A7r5 cells. These results suggest that the changes of ADM and its receptor system may exert beneficial effects on blood vessels in OH state. RAMP2 and RAMP3 were the key determinants for biological activities of ADM. The CRLR combining with RAMP 2 or 3 confers specificity of the receptor for ADM [30]. In this study, we applied the ADM receptor antagonist and found that it markedly blocked the inhibitory effects of ADM on PA or PA plus Ang II-induced inflammation, oxidative stress and calcification in A7r5 cells suggesting that ADM may be through the receptor activation to exert its protective roles in vascular diseases in OH state.

AMP-activated protein kinase (AMPK), as a sensor of energy molecules, is closely associated with metabolic stress, diabetes and vascular disorders [31,32,33,34,35,36]. In VSMCs, AMPK activation also involves the improvement of oxidative stress, inflammation and calcification [37,38]. Both intermedin (IMD), also known as adrenomedullin 2 (ADM2), and ADM belong to the calcitonin/calcitonin gene-related peptide family and they exert some similar functions in some organs or cells through similar mechanisms [39]. IMD can activate AMPK to alleviate insulin resistance, vascular calcification and cardiac hypertrophy [40,41,42]. Thus, we speculate that the protective roles of ADM in this study may be through the activation of AMPK. In vitro experiments, we applied the AMPK inhibitor Compound C to confirm the hypothesis. The results showed that Compound C pretreatment indeed attenuated the protective roles of ADM in PA or PA plus Ang II-induced inflammation, oxidative stress and calcification in A7r5 cells indicating that AMPK activation may partly involve the beneficial roles of ADM in vascular diseases in OH state. In this study, the intracellular signaling pathway of ADM remains mostly to be specified. For instance, we can further measure cAMP level, a major intracellular messenger of ADM and apply the cAMP analogue to mimic ADM’s actions that further clarifies the mechanisms of ADM’s roles in OH.

Based on the results of the present study, we concluded that ADM could effectively improve hypertension, vascular remodeling and arterial stiffness in OH rats, which might be closely associated with the inhibition of inflammation, oxidative stress and calcification in VSMCs partially via the receptor-mediated AMPK pathway. However, the precise mechanism underlying ADM’s action in this study needs to be further investigated. 

## Data Availability

The data of this study will be available from the authors according to reasonable requests.
